# Assessment of neonatal cerebral oxygenation using near-infrared spectroscopy in cesarean sections performed with neuraxial blocks: a prospective randomized clinical trial

**DOI:** 10.1590/1806-9282.20250678

**Published:** 2025-12-15

**Authors:** Ahsen Gur Celık, Derya Karasu, Nermin Kılıçarslan, Canan Yılmaz, Aycan Kurtarangıl Dogan, Gurcan Guler, Seyda Efsun Ozgunay, Mehmet Gamlı

**Affiliations:** 1 Bursa Orhangazi State Hospital, Department of Anaesthesiology and Reanimation – Bursa, Turkey.; 2 University of Health Sciences Turkey, Bursa Yuksek Ihtisas Training and Research Hospital, Department of Anaesthesiology and Reanimation – Bursa, Turkey.; 3 Kütahya City Hospital, Department of Anaesthesiology and Reanimation – Kütahya, Turkey.

**Keywords:** Spinal anesthesia, Epidural anesthesia, Cesarean section, Newborn, Near-infrared spectroscopy

## Abstract

**OBJECTIVE:**

The aim of the study was to evaluate the effects of epidural and spinal anesthesia techniques on neonatal cerebral oxygenation during cesarean surgery using near-infrared spectroscopy.

**METHODS:**

Pregnant women aged 18–35 years, classified as American Society of Anesthesiologists (ASA) II, scheduled for elective cesarean surgery at >37 weeks of gestation, and their newborns were included. Two groups, one to receive spinal anesthesia (Group S) and the other epidural anesthesia (Group E), were determined. Appearance Pulse Grimace Acitivity Respiration (APGAR) scores, heart rates, and SpO_2_ values of the newborns were recorded, and umbilical venous blood gas was collected. Near-infrared spectroscopy and rSO_2_ (regional cerebral oxygen saturation) change percentage were measured.

**RESULTS:**

A total of 80 patients were statistically analyzed, with 40 patients in Group S and 40 patients in Group E. Neonatal SpO_2_ values at 2.5 min were found to be significantly lower in Group S, while in blood gas parameters, PaO_2_ values were significantly lower in Group S. Near-infrared spectroscopy values at 2.5 and 5 min were significantly lower in Group S, and rSO_2_ change percentages were significantly lower at 2.5 min in Group S. Maternal hypotension was observed to be significantly higher in Group S (52.5%) than in Group E (22.5%). The amount of ephedrine administered until delivery was higher in Group S, but this difference was not statistically significant between the two groups.

**CONCLUSION:**

We found significantly lower near-infrared spectroscopy values at 2.5 and 5 min and rSO_2_ change percentage at 2.5 min in the spinal group. We believe that epidural anesthesia has minimally more positive effects on early neonatal cerebral oxygenation compared to spinal anesthesia.

## INTRODUCTION

Regional anesthesia techniques are more commonly used in cesarean section anesthesia. Hypotension due to sympathetic system blockade is a frequent finding in neuraxial blocks. The incidence of hypotension in cesarean sections ranges between 50 and 80%^1^. The incidence of maternal hypotension has been reported as 24–53% in epidural anesthesia^2,3^ and over 80% in spinal anesthesia^3,4^. Hypotension causes symptoms such as nausea, vomiting, and dizziness in the mother, while maternal hypotension leads to hypoxia, acidosis, loss of tone, and other findings in the newborn. Preventing hypotension in pregnant women and intervening quickly when it occurs is crucial in avoiding fetal complications.

After birth, the newborn's arterial oxygen saturation is expected to be above 80% at 5 min and above 90% at 10 min. To assess the newborn's transition status, peripheral oxygen saturation (SpO_2_) and regional cerebral oxygen saturation (rSO_2_) measurements can assist clinicians^5^. To evaluate the adequacy of cerebral perfusion, a low-cost, non-invasive, and portable near-infrared spectroscopy (NIRS) technology is used. Its mechanism is based on the selective transmission of light photons in the infrared spectrum with a wavelength of 700–850 nm as they pass through tissues and the selective absorption of these photons by chromophores (hemoglobin, myoglobin, and cytochromes). This technique is used to estimate rSO_2_ values in tissues. NIRS allows real-time and continuous estimation of regional tissue oxygenation^6–10^.

The aim of our study is to evaluate the effects of epidural and spinal anesthesia on neonatal cerebral oxygenation after elective cesarean sections using NIRS.

## METHODS

Ethical approval for this study was obtained from the local ethics committee and Clinical Trial Registry (NCT06316596). The NIRS probes (Medtronic INVOS 7100 Oximeter) used for our study were funded by our hospital's scientific research support fund. This prospective, randomized, and single-blind study was conducted on 90 patients scheduled for cesarean section at Bursa Yuksek Ihtisas Training and Research Hospital in Turkey. Informed consent was obtained from all patients. Patients aged 18–35 years, classified as ASA II, with a gestational age of >37 weeks undergoing elective cesarean section, and their newborns were included in the study. Patients with allergies to the medications used, bleeding diathesis, mental disorders, those who did not consent to participate, patients with a height <1.55 or >1.70 m, those with infections at the block site, a body mass index (BMI) >35 kg/m², pregnancy-induced hypertension, or pre-pregnancy systolic blood pressure >140 mmHg or diastolic blood pressure >90 mmHg in three measurements, known fetal anomalies, placental pathologies, or a history of fetal anomalies or abnormal deliveries in previous pregnancies were excluded from the study. Patients who failed neuraxial anesthesia and required general anesthesia, those with fetal distress, newborns born with meconium aspiration, newborns requiring resuscitation, and those with difficulties in NIRS monitoring were also excluded.

Randomization of the patients was performed using computer-generated randomization. Group S (n=40) consisted of patients who received spinal anesthesia, and Group E (n=40) consisted of those who received epidural anesthesia. To ensure the quality and standardization of the block, both spinal and epidural anesthesia were administered by an experienced anesthesiologist. Surgeries were performed by the same surgical team.

Demographic data of the patients were recorded, and routine non-invasive monitoring was performed.

Spinal anesthesia was performed in the left lateral decubitus position (25G Quincke needle, L_3–4_/L_4–5_ level). A dose of 10 mg of 0.5% hyperbaric bupivacaine was administered. The individual was subsequently positioned supine, and a pinprick assessment was conducted to evaluate sensory perception. Spinal level checks were conducted, and a sensory block level up to T_5_ was considered adequate for surgery.

Epidural anesthesia was performed in the left lateral decubitus position; the epidural space was localized at either the L_3–4_ /L_4–5_ vertebral interspace utilizing a Tuohy needle and the loss-of-resistance method. An epidural catheter was subsequently advanced 3–5 cm into the identified epidural space. A test dose was administered. A solution of 15–20 mL was administered through the epidural catheter (20 mL solution=10 mL 0.5% bupivacaine+1 mL 50 mcg fentanyl+5 mL 2% lidocaine+4 mL normal saline). A sensory block level up to T_5_ was considered adequate for surgery. If bilateral blockade at the T_6_ level was not achieved within 10 min after the initial dose, additional 5 mL increments of 0.25% bupivacaine were administered epidurally every 5 min, up to a maximum cumulative dose of 30 mL. If the epidural block did not reach the T_5–6_ level within 20 min, the patient was planned to be switched to general anesthesia and excluded from the study.

In both groups, the time of initiation of regional anesthesia was recorded, and the period until skin incision was considered the preparation phase for surgery. Maternal vital signs were recorded at 3-min intervals from the preparation phase until the delivery of the newborn. If the mean arterial pressure (MAP) decreased by 25% or more compared to the baseline value, intravenous ephedrine was administered. If the heart rate decreased by 25% or more, intravenous atropine was administered. The time from skin incision to delivery of the baby was recorded as the cord clamping time. Maternal side effects were recorded.

In both groups, the newborn was placed on a pre-warmed radiant heater after delivery, and routine care was provided as standard. An anesthesiologist (blinded to the anesthesia technique) other than the one performing the neuraxial block attached an NIRS probe to the newborn's frontal region and a transcutaneous pulse oximeter (SpO_2_) to the right hand. The same anesthesiologist recorded the measurements. Umbilical venous blood gas was collected. APGAR scores, heart rate, and SpO_2_ values of the newborn were recorded. NIRS measurements, rSO_2_ change percentages, and SpO_2_ values were assessed.

### Statistical analysis

Data were analyzed using Statistical Package for the Social Sciences (SPSS)-25.0. Categorical data were summarized using frequencies and percentages. Continuous variables were reported as means, standard deviations, medians, and ranges (minimum–maximum). For assessing differences in continuous variables between two independent groups, the independent samples t-test and Mann-Whitney U test were applied. For comparisons of qualitative variables between independent groups, chi-square tests were employed. The required sample size for the study was calculated based on an effect size of 0.56 for the independent samples t-test, with 80% test power and a 95% confidence level, resulting in a minimum sample size of 80 participants. A p-value of less than 0.05 was considered statistically significant.

## RESULTS

Out of 90 patients who received regional anesthesia for cesarean delivery, 81 were included in the study. In Group E, one patient was excluded due to difficulties in NIRS monitoring. Statistical analysis was performed on a total of 80 patients. There were no significant differences between the groups in terms of patients’ demographic data (Table 1). The incidence of hypotension was significantly higher in Group S (52.5%). The administration of atropine was not required in any of the patients. However, supplemental dosing was necessary in 10 patients within Group E. No statistically significant differences were identified among the groups with respect to neonatal demographic characteristics (Table 1).

**Table 1:** Demographic data of the patients.

		Group S (n=40)	Group E (n=40)	p
Age (years), mean±SD		28.18±6.30	29.73±5.76	0.254^a^
BMI (kg/m²), mean±SD		29.79±4.97	29.74±4.51	0.966^a^
Gestational age (days), mean±SD		265.7±5.68	266.50±4.46	0.134^b^
**Obstetric history, n (%)**				
	• Primiparous	14 (35.0)	13 (32.5)	1.000^b^
	• Multiparous	26 (65.0)	27 (67.5)	
Smoking status, n (%)		3 (7.5)	6 (15.0)	0.481^a^
The time from skin incision to delivery, minutes		6.75±2.70	7.48±3.37	0.292^b^
Frequency of hypotension, n (%)		21 (52.5)	9 (22.5)	**0.011** c
Duration of hypotension (seconds)		90.42±54.09	62.50±15.45	0.295^b^
**Maternal complications, n (%)**				
	Nausea	14 (35.0)	7 (17.5)	0.127^c^
**Neonatal**				
**Gender, n (%)**				0.371^c^
	• Male	22 (55.0)	18 (45.0)	
	• Female	18 (45.0)	22 (55.0)	
Weight (grams), mean±SD		3,096.8±427.9	3,232.0±405.2	0.108^b^
APGAR scores at 1, 5, and 10 min >7		40 (100)	40 (100)	

SD: standard deviation; BMI: body mass index; APGAR: Appearance Pulse Grimace Acitivity Respiration;

a Fisher chi-square test;

b Yates chi-square test;

c Mann-Whitney U test.

Statistically significant p-values are indicated in bold.

No statistically significant differences were noted between the groups regarding neonatal heart rate measurements (Table 2). However, the 2.5-min SpO_2_ values and umbilical cord blood gas analysis results, particularly the PaO_2_ levels, were significantly lower in Group S compared to the other group. None of the newborns required positive pressure ventilation (PBV) or intubation. Additionally, none of the newborns in either group required intensive care within the first 24 h.

**Table 2:** Neonatal heart rate and peripheral oxygen saturation values (mean ± SD).

		Group S (n=40)	Group E (n=40)	p
Heart rate	1 min	167.1±14.5	163.2±18.8	0.704^b^
	2.5 min	165.0±13.2	161.6±12.3	0.238^a^
	5 min	161.8±14.1	160.7±11.7	0.699^a^
	10 min	162.2±13.1	161.2±12.9	0.725^a^
SpO_2_	1 min	76.9±8.4	79.8±8.4	0.228^b^
	2.5 min	83.0±5.2	87.7±5.2	**0.002** a
	5 min	88.6±4.9	90.7±4.9	0.066^a^
	10 min	93.0±3.7	94.5±3.7	0.056^a^
Ph		7.33±0.03	7.3±0.05	0.445^b^
PaO_2_ (mmHg)		20.3±7.2	25.4±8.2	**0.005** a

SpO_2_: peripheral oxygen saturation.

a Fisher chi-square test;

b Yates chi-square test.

Statistically significant p-values are indicated in bold.

The NIRS values and rSO_2_ change percentages according to the groups are presented in Table 3. The 2.5- and 5-min NIRS values, as well as the 2.5-min rSO_2_ change values, were significantly lower in Group S (p<0.05). When the correlation of the NIRS decrease at these time points with parameters such as maternal age, BMI, smoking status, neonatal weight, presence and duration of maternal hypotension, and clamp time was examined, only the presence of hypotension was found to be significantly associated with lower NIRS values (p=0.009).

**Table 3:** NIRS values (mean±SD) and rSO_2_ changes in the groups.

		Group S (n=40)	Group E (n=40)	p
NIRS	1 min	57.4±16.4	59.0±10.9	0.912^a^
	2.5 min	66.8±14.4	75.8±10.0	**0.012** a
	5 min	79.3±10.2	83.8±8.7	**0.049** a
	10 min	84.5±8.3	86.0±7.3	0.490^a^
rSO_2_ (%)	1–2.5 min	16.00	29.00	**0.025**
	1–5 min	37.00	48.00	0.564
	1–10 min	44.00	50.00	0.942

NIRS: near infrared spectroscopy, rSO_2_: regional cerebral oxygen saturation.

a Mann-Whitney U test.

Statistically significant p-values are indicated in bold.

## DISCUSSION

Neuroaxial anesthesia techniques are frequently used in cesarean deliveries today. One of the most common complications is hypotension, which, if prolonged and not promptly managed, can affect uteroplacental blood flow, leading to fetal hypoxia, acidosis, and low APGAR scores. In recent years, the increasing use of NIRS monitoring and rSO_2_ measurements provides information on hemoglobin oxygenation and the saturation of the brain and other organs. In our prospective randomized study, pregnant women and their newborns undergoing elective cesarean delivery under spinal or epidural anesthesia were evaluated. In addition to standard neonatal monitoring, NIRS monitoring was performed. Maternal hypotension until delivery was significantly more frequent in Group S (52.5%) compared to Group E (22.5%). No statistically significant differences were detected in the amount of ephedrine administered or the duration of hypotension. Neonatal PaO_2_ levels and 2.5-min SpO_2_ values were significantly lower in Group S. Additionally, the 2.5- and 5-min NIRS values, as well as the 2.5-min rSO_2_ change percentage, were significantly lower in Group S.

In the study by Urlesberger et al.^11^ changes in rSO_2_ in term newborns after cesarean and vaginal delivery were recorded. The SpO_2_ values between the 4th and 8th min were significantly lower in cesarean deliveries compared to vaginal deliveries, and the heart rate values of newborns delivered by cesarean were lower throughout the monitoring period. Although no statistically significant differences were noted in rSO_2_ values between the groups, heart rate and SpO_2_ were significantly lower in the cesarean group, while no significant difference in rSO_2_ values was found based on the mode of delivery. In the study by Ozgen et al.^5^ which examined pregnant women undergoing combined spinal-epidural and general anesthesia, the SpO_2_ and rSO_2_ values (right-left frontal measurements) of newborns between the 1st and 2.5 min were evaluated. They reported that both SpO_2_ and rSO_2_ values were elevated in the combined spinal-epidural group. In our study, the 2.5-min SpO_2_ values and the percentage change in rSO_2_ at the 2.5-min mark were significantly lower in the spinal anesthesia group when compared to the epidural anesthesia group.

Berlac et al.^1^ investigated the predictability of hypotension using the NIRS method during spinal anesthesia in elective cesarean sections. In their study of 35 patients, they demonstrated that in 22 pregnant women, there was a reduction of more than 5% in rSO_2_ before the onset of symptoms or hypotension. At the same time, 2 out of the 13 pregnant women who did not develop hypotension also showed a reduction in rSO_2_ of more than 5%.

Whether rSO_2_ significantly differs between various brain regions has not been sufficiently clarified in the literature. Lemmers et al.^12^ demonstrated that there could be differences between the left and right hemispheres, particularly in cases of unstable arterial saturation. For this reason, we used the same frontotemporal region in all infants in our study.

In the review of NIRS in neonates at high risk of impaired perfusion, tissue oximetry has been proposed as a non-invasive method for the continuous monitoring and early detection of hypoperfusion states. Although NIRS technology has limitations in precisely correlating with validated and directly measured parameters of systemic oxygen delivery and blood flow, it has been suggested that NIRS may aid in identifying low cardiac output. However, to more clearly establish its clinical utility, prospective, randomized, and rigorously designed studies are recommended.^13^ In our study, neonates with congenital anomalies were excluded; consequently, low cardiac output states resulting from congenital defects could not be assessed.

The limitation of our study is that NIRS monitoring was evaluated solely through a single frontal region, which restricts the comprehensive assessment of oxygenation. Additionally, the impact of low NIRS values on the length of hospital stay, as well as long-term respiratory and neurological outcomes in neonates, was not evaluated. Other limitations include the absence of maternal NIRS monitoring and the use of non-invasive techniques for blood pressure assessment.

The 2.5-min SpO_2_ and PaO_2_ values in neonates were significantly lower in Group S. Similarly, the 2.5- and 5-min NIRS values, as well as the 2.5-min rSO_2_ percentage change, were significantly lower in Group S. In conclusion, we suggest that epidural anesthesia has a slightly more favorable effect on neonatal cerebral oxygenation in the early period compared to spinal anesthesia, albeit minimal. We believe that further multicenter, prospective, and randomized controlled studies are needed to explore this topic in greater depth.

## Data Availability

The datasets generated and/or analyzed during the current study are available from the corresponding author upon reasonable request.
